# OMAL: A Multi-Label Active Learning Approach from Data Streams

**DOI:** 10.3390/e27040363

**Published:** 2025-03-29

**Authors:** Qiao Fang, Chen Xiang, Jicong Duan, Benallal Soufiyan, Changbin Shao, Xibei Yang, Sen Xu, Hualong Yu

**Affiliations:** 1 School of Computer, Jiangsu University of Science and Technology, Zhenjiang 212100, China; fangqiao@stu.just.edu.cn (Q.F.); chen_xiang@stu.just.edu.cn (C.X.); jicong_duan@stu.just.edu.cn (J.D.); benallalsoufiane1@gmail.com (B.S.); shaocb@just.edu.cn (C.S.); jsjxy_yxb@just.edu.cn (X.Y.); 2 School of Information Technology, Yancheng Institute of Technology, Yancheng 224051, China; xusen@ycit.edu.cn

**Keywords:** active learning, multi-label data stream, query strategy, classifier chains, weighted extreme learning machine, label correlations, class imbalance learning

## Abstract

With the rapid growth of digital computing, communication, and storage devices applied in various real-world scenarios, more and more data have been collected and stored to drive the development of machine learning techniques. It is also noted that the data that emerge in real-world applications tend to become more complex. In this study, we regard a complex data type, i.e., multi-label data, acquired with a time constraint in a dynamic online scenario. Under such conditions, constructing a learning model has to face two challenges: it requires dynamically adapting the variances in label correlations and imbalanced data distributions and it requires more labeling consumptions. To solve these two issues, we propose a novel online multi-label active learning (OMAL) algorithm that considers simultaneously adopting uncertainty (using the average entropy of prediction probabilities) and diversity (using the average cosine distance between feature vectors) as an active query strategy. Specifically, to focus on label correlations, we use a classifier chain (CC) as the multi-label learning model and design a label co-occurrence ranking strategy to arrange label sequence in CC. To adapt the naturally imbalanced distribution of the multi-label data, we select weight extreme learning machine (WELM) as the basic binary-class classifier in CC. The experimental results on ten benchmark multi-label datasets that were transformed into streams show that our proposed method is superior to several popular static multi-label active learning algorithms in terms of both the Macro-F1 and Micro-F1 metrics, indicating its specifical adaptions in the dynamic data stream environment.

## 1. Introduction

In recent years, active learning has been developed to be a significant machine learning paradigm for specific application in a scenario where it is easy to collect an amount of instances, but labeling them requires unacceptable time, human, and economic costs [1,2]. In such a scenario, active learning can best improve the performance of a learning model by iteratively querying a few significant instances from human annotators (oracles). Some previous studies [3,4,5] have indicated that by active learning, a learning model can acquire comparative or better performance to a model trained with labeling all instances.

**Figure 1:**
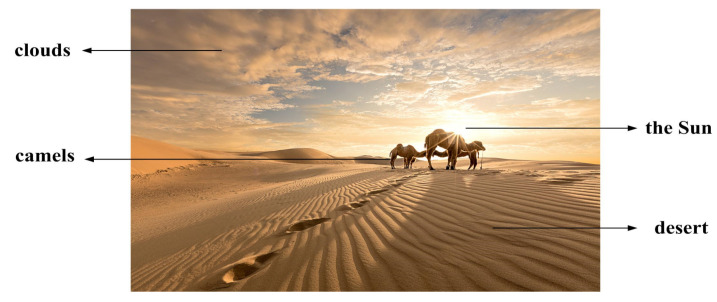
An example of a multi-label image.

We note that most existing active learning studies focused on single-label data [3,4,5,6,7], i.e., each instance is only associated with a specific label. However, in many real-world applications, data may simultaneously associate with multiple labels, e.g., an image may contain desert, camels, clouds, and the Sun (see Figure 1); a news article may cover several different topics, such as war, economics, and politics; and a movie may relate to more than one topic, such as comedy, cartoon, and action. In general, we refer to this type of data as multi-label data, which widely exist in various real-world applications, including image recognition [8], recommender system [9,10], text classification [11], disease diagnosis [12], and sensor data analysis [13,14]. For multi-label data, learning models are required to simultaneously label all labels for an instance; thus, it is obviously more difficult to evaluate the significance level of an unlabeled multi-label instance than a single-label one in active learning [15].

Several studies have focused on multi-label active learning issues where the core concern lies in how to design a query strategy for determining which unlabeled instances are important for significantly improving the performance of learning models. In previous studies, *uncertainty* [16] and *diversity* [17] have been the two most widely used criteria for designing query strategies. Specifically, the *uncertainty* criterion always searches instances that are close to the classification boundary of the current learning model, while the *diversity* criterion focuses on the deviation level between unlabeled instances and the instances in the labeled set. Representative multi-label active learning methods include example-based active learning (EMAL) [18], label cardinality inconsistency (LCI) [19], and category vector inconsistency and ranking of scores (CVIRS) [20]. EMAL only adopts the *uncertainty* criterion to select significant unlabeled instances. Specifically, it first independently measures the uncertainty level in each label by the traditional single-label uncertainty level detection strategy, and then it uses the sum of these uncertainty levels to evaluate the uncertainty level in the corresponding multi-label instance. LCI regards the unlabeled instance whose number of pseudo-labels predicted by the current learning model has the most significant difference with the average label cardinality of all labeled instances as the most uncertain one. CVIRS focuses on both *uncertainty* and *diversity*, where the uncertainty level is measured by ranking the distances between all unlabeled instances and the current classification boundary, and the diversity level is quantized by calculating the average difference between the predicted label vector of an unlabeled instance and the real label vectors of all labeled instances.

Although there are some multi-label active learning methods, most of them only focus on static scenarios, i.e., all unlabeled instances are previously available. However, in many real-world applications, data can only be collected with a time constraint, i.e., emerging in the form of a data stream [21,22,23]. In such a scenario, considering the potential unlimitedness of the data stream and the finiteness of the data store, it is inevitable that the learning model must abide by the *one pass* rule [24]. This means that when a new unlabeled data chunk is received, the active learning model is required to immediately decide which instances are important and should be labeled and stored and then remove all other instances, which cannot be revisited. The active learning algorithms based on such a constraint are expected to simultaneously improve the quality of the learning model and adapt to potential concept drift [25,26,27]. To our best knowledge, only one previous work partially focuses on such a learning scenario and proposes an ensemble learning solution [28]. However, it has two significant drawbacks, as follows: (1) it independently trains a KNN classifier for each label, thus failing to focus on label correlations [29,30] that have been proven to be important for training multi-label learning models, and (2) it neglects the naturally imbalanced issue existing in multi-label data [31,32]. Therefore, it is necessary to design some effective and efficient multi-label active learning algorithms for application in the data stream environment.

For the purpose mentioned above, a novel online multi-label active learning algorithm, called OMAL, is proposed in this study. Similar to CVIRS [20], OMAL simultaneously focuses on both the *uncertainty* and *diversity* criteria within its query strategy. Specifically, the uncertainty level of an unlabeled instance is measured by calculating the average entropy of the prediction probability provided by the learning model across all labels. As we know, the entropy of the prediction probability can reflect how close an instance is the classification boundary. As for the *diversity* criterion, we do not consider the strategy of CVIRS but adopt the average cosine distance between the feature vector of an unlabeled instance and all labeled instances to calculate it. In contrast to calculating the difference between label vectors, such a strategy can more directly discover those diverse unlabeled instances. In the OMAL query strategy, the significance level of an unlabeled instance is integrated by weighting its uncertainty and diversity levels. In addition, considering that label dependency and a naturally imbalanced data distribution always exist in multi-label data, we adopt a classifier chain (CC) [33,34] as the classification model, in which a label co-occurrence ranking strategy is embedded to arrange the label sequence and guarantee the performance of classification models. We also use weighted extreme learning machine (WELM) [35] as the basic binary classification model for adapting the imbalanced data distribution. The experimental results on ten benchmark multi-label datasets that were transformed to be streams show that our proposed method is superior to several popular static multi-label active learning algorithms in terms of both the Macro-F1 and Micro-F1 metrics, indicating its effectiveness and superiority.

Specifically, the novelties of this study are listed as follows:

1. A novel instance significance active query strategy, which simultaneously considers *uncertainty* and *diversity*, is proposed;
2. To adapt both requirements of leveraging label correlations and treating class imbalance in multi-label learning, CC and WELM learning models are integrated into an online active learning framework;
3. A novel multi-label online active learning algorithm, called OMAL, is proposed in this study, and to our best knowledge, it is the first algorithm that totally considers all requirements in this specific learning scenario.

The rest of this paper is organized as follows. Section 2 first simply describes the basic framework of stream-based active learning; then, it introduces the query strategy and classification models in detail and, finally, presents the flow path of the proposed OMAL algorithm and discusses its time complexity. In Section 3, the experimental results and analysis are provided in detail. Finally, Section 4 discusses the findings and contributions of this study and further indicates the future work.

## 2. Methods

### 2.1. The Basic Framework of Stream-Based Active Learning

**Figure 2:**
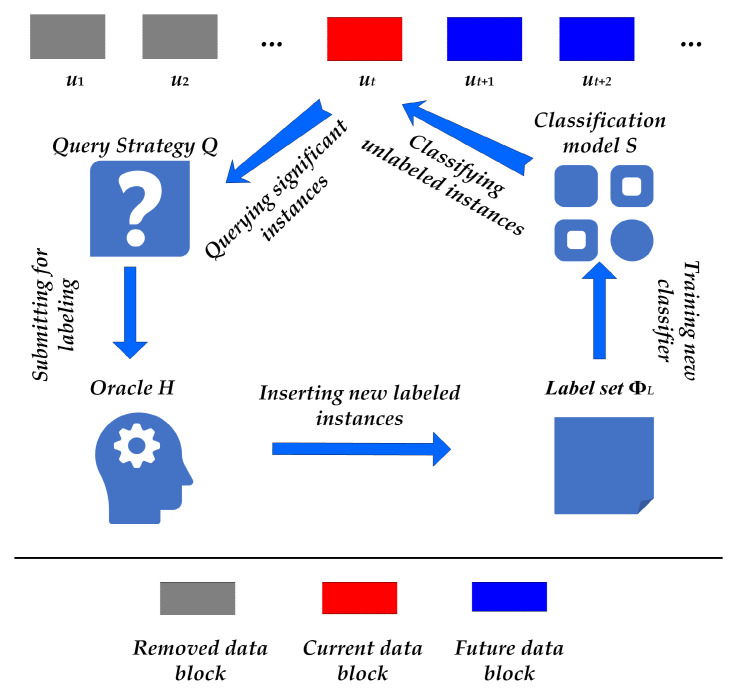
The basic framework of stream-based active learning.

In a stream-based active learning framework, there are generally five basic components, as follows: a labeled set $\Phi_{L}$, a most recently received unlabeled set $U_{t}$, a classification model *S*, a pre-designed query strategy *Q*, and an oracle *H*, which may be either a human annotator or others, e.g., ChatGPT. During active learning, when a new unlabeled data chunk $U_{t}$ is received, the query strategy *Q* and classification model *S* are both activated to select some significant instances from $U_{t}$ to be submitted to the oracle *H* for labeling. Next, this new labeled set $U_{t}^{\prime }$, where $U_{t}^{\prime }\subseteq U_{t}$, is added into the labeled set $\Phi_{L}$, i.e., $\Phi_{L}=\Phi_{L}\cup U_{t}^{\prime }$, to be used for training a new classifier to replace *S*. The aforementioned procedure is iteratively conducted while continually receiving new unlabeled data. The basic framework is described in Figure 2. Specifically, when data simultaneously associate with multiple labels, both the classification model *S* and the query strategy *Q* are required to adapt to the multi-label scenario. The differences between pool-based active learning and stream-based active learning are reflected in the two following aspects: (1) in static active learning, *Q* can directly visit the whole unlabeled set *U*, while online active learning can only visit the current unlabeled chunk $U_{t}$, and (2) static active learning reserves all unlabeled instances $U=U\backslash U_{t}^{\prime }$ after each iteration, while online active learning abandons all remaining unlabeled instances $U_{t}^{\prime \prime }=U_{t}\backslash U_{t}^{\prime }$ in the current data chunk to hinder revisiting forever.

### 2.2. Query Strategy of OMAL

Suppose that $\Phi_{L}=\{\left(x_{i},Y_{i}\right)|x_{i}\in R^{m},i\in \left\{1,2,\ldots ,n\right\},Y_{i}=\left[y_{i1},y_{i2},\ldots ,y_{i\left|L\right|}\right]\}$ is a labeled multi-label set and $u_{t}=\{x_{j}|x_{j}\in R^{m},j\in \left\{1,2,\ldots ,k\right\}\}$ is a new received unlabeled block, where $x_{i}$ and $Y_{i}$ respectively denote a feature vector and a label vector corresponding to the ith instance, n and k respectively represent the number of instances in the labeled set and new received unlabeled block, $y_{ik}$ indicates the kth class label of the ith instance, m denotes the number of features, and |*L*| represents the number of labels. Active learning is required to extract *k′* (*k′* < *k*) instances, according to a pre-designed query strategy Q, from $u_{t}$ for submission to the oracle H for labeling, which are then used to extend $\Phi_{L}$. For an active learning algorithm, its query strategy is most important as it directly relates to the quality of the algorithm.

In this study, we combine both the *uncertainty* and *diversity* criteria to design a multi-label query strategy. As indicated in Section 1, the *uncertainty* criterion aims to search those instances that are most close to the current classification boundary. These instances often emerge in the class overlapping area, and thus, their label confidences are much lower than those of other instances. We use the average entropy of the prediction probability across all |*L*| labels to search close-to-boundary instances. The difficulty lies in identifying how to calculate the prediction probability for a non-Bayes classification model. Fortunately, for our selected WELM classifier, there is a way to transform the soft outputs into prediction probabilities [36]. Suppose that for an unlabeled instance $x_{j}$, its output in the trained WELM corresponding to the *i*th label is $f_{i}(x_{j})$; then, it can be transformed as a prediction probability $P(y_{i}|f_{i}(x_{j}))$ by the sigmoid function as follows:

(1) $$P\left(y_{i}|f_{i}\left(x_{j}\right)\right)=\frac{1}{1+exp(-f_{i}(x_{j}))}$$

It has been shown that the transformation strategy can create an approximately accurate mapping between the outputs of extreme learning machine and posterior probabilities of the naïve Bayes classifier in theory [36]. Based on this transformation, the average entropy of $x_{j}$ can be calculated as follows:

(2) $$EN\left(x_{j}\right)=-\frac{1}{\left|L\right|}\sum_{i=1}^{\left|l\right|}\;\left[P\left(y_{i}|f_{i}\left(x_{j}\right)\right)\log P\left(y_{i}|f_{i}\left(x_{j}\right)\right)+\left(1-P\left(y_{i}|f_{i}\left(x_{j}\right)\right)\right)\log\left(1-P\left(y_{i}|f_{i}\left(x_{j}\right)\right)\right)\right]$$

where $EN\left(x_{j}\right)$ denotes the average entropy of the instance $x_{j}$ across all labels. It is clear that if an instance has a larger *EN*, it will be more uncertain.

As for the *diversity* criterion, we consider that if two instances have a more approximated distribution in the feature space, then it might mean that their labels are more similar. In the same fashion, when an instance has a large difference from all other instances, it may represent a novel pattern. Based on this basic hypothesis, we suggest adopting the average cosine distance between an unlabeled instance $x_{j}$ and all *n* labeled instances in $\Phi_{L}$ to calculate the *diversity* of $x_{j}$. The reason for adopting the cosine distance but not the Euclidean distance is that the cosine distance focuses more on the direction difference between two instances, and thus, it is more robust than the Euclidean distance, especially when the data are highly dimensional. For an unlabeled instance $x_{j}$ and any one labeled instance $x_{i}$, their cosine distance is calculated as follows:

(3) $$\cos\left(x_{i},x_{j}\right)=\frac{x_{i}\cdot x_{j}}{\left|\left|x_{i}\right|\right|\;||x_{j}||}$$

where $x_{i}\cdot x_{j}$ represents their dot product and $\left|\left|x_{i}\right|\right|$ denotes the norm of the vector $x_{i}$. However, we note that when the cosine distance between two instances is larger, their difference is smaller, which is inconsistent with the requirement of diversity evaluation. Thus, we define the diversity measure as follows:

(4) $$d\left(x_{j}\right)=1-\frac{1}{n}\sum_{i=1}^{n}\cos\left(x_{i},x_{j}\right)$$

where $d\left(x_{j}\right)$ denotes the diversity level of $x_{j}$ corresponding to all *n* labeled instances. Selecting those unlabeled instances with a large diversity to the labeling instances helps to cover diverse instance patterns, further improving the robustness of the classification models.

In our proposed query strategy, the significance level of an unlabeled instance can be calculated by weighting both the uncertainty level and diversity level as follows:

(5) $$s\left(x_{j}\right)=\lambda \times EN\left(x_{j}\right)+(1-\lambda )\times d\left(x_{j}\right)$$

where $s\left(x_{j}\right)$ denotes the significance level of the unlabeled instance $x_{j}$ and λ (λ∈[0,1]) is the weighing factor that is used to regulate the relative significance of *uncertainty* and *diversity*. In this study, we empirically designate λ as 0.5, i.e., *uncertainty* and *diversity* contribute equally to the significance level.

### 2.3. Classification Models Used in OMAL

#### 2.3.1. Classifier Chains

It is well known that the reason why it is more difficult to construct a multi-label learning model than a single-label one lies in the fact that some label correlations exist in multi-label data [29,30], e.g., if an image has the *sandbeach* label, then it has a higher probability of simultaneously owning the *sea* label but a lower probability of holding the *waterfall* label. Therefore, it is necessary to make multi-label classification models leverage label correlation information. CC [33,34] belongs to this kind of learning model, and it has been widely used in many real-world multi-label applications. CC first arranges all |*L*| labels into a chain as follows: $l_{1}\to l_{2}\to \cdots \to l_{\left|L\right|}$. It then sequentially trains the |*L*| binary-class classifier to predict each label. Specifically, for the *i*th learning model, the feature space of training instances is extended to include both original features and the first *i* − 1 labels. Suppose the original feature space is represented by *X*; then, the first binary classifier can be represented as $CL(X)\to l_{1}$, and the second binary classifier can be represented as $CL(X\cup l_{1})\to l_{2}$. In a similar fashion, the final binary classifier can be represented as $CL(X\cup l_{1}\cup l_{2}\cup \ldots \cup l_{\left|L-1\right|})\to l_{\left|L\right|}$, where CL denotes a binary classifier. By this way, class correlations are partially embedded into the learning model *S*, helping to improve its prediction performance.

However, for CC, some previous studies have found that the order of labels in sequence can directly influence the performance of the learning model [37,38]. If some difficult labels are first predicted, then the error accumulation phenomenon would emerge to misguide the prediction of subsequent labels. Aiming to address this issue, we used the idea of label dependency drift detector (LD3) [39] to design a label significance ranking strategy. Specifically, LD3 was originally proposed to detect label matching level between two data chunks existing in a multi-label data stream. In our algorithm, we only take advantage of the label correlation significance ranking strategy adopted by LD3. For the labeled set $\Phi_{L}$, it first calculates the co-occurrence matrix, which is obtained by counting the number of times each class label occurs as “1” alongside other labels. The generated matrix is then ranked within each row, which is called local ranking, by creating a ranking for each label based on their co-occurrence frequencies. After acquiring the local ranking, the ranks can be further aggregated as the global ranking by

(6) $$r_{i}=\frac{1}{\sum_{j=1,j\neq i}^{\left|L\right|}\frac{1}{r_{ij}}}$$

where $r_{i}$ denotes the ranking score of the label $l_{i}$ and $r_{ij}$ represents the ranking of the label $l_{i}$ in the *j*th row of the local ranking matrix. Next, we obtain the global ranking position sequence *R* by ranking all $r_{i}$ in ascending ranking. Obviously, the labels with more correlations with other labels will have a higher ranking in *R*, and predicting these labels first could provide more useful information for improving the accuracy of other labels.

#### 2.3.2. Weighted Extreme Learning Machine

As we know, multi-label data always suffer from the class imbalance problem on most or all of the labels [31,32]; thus, we should not ignore this issue when selecting the basic binary classification model. For the binary-class imbalanced issue, there are abundant solutions, including sampling [40], cost-sensitive learning [35], threshold moving [41], and ensemble learning [42,43]. Considering the time-complexity requirement of the online environment, we decided to adopt an efficient, cost-sensitive learning method, that is, weighted extreme learning machine (WELM), which is both fast and robust [35].

WELM is a variant of ELM [44,45], aiming to alleviate the impact of imbalanced data distributions. ELM is a fast learning algorithm used to train single-hidden-layer feedforward neural networks (SLFNs). For a binary-class problem, let us suppose there are *n* training instances ($x_{i},\;t_{i}$), where $x_{i}\in R^{m}$ and $t_{i}\in \{-1,1\}$. If an SLFNs with *L* hidden nodes can approximate these *n* instances with zero error, then it implies that there exists $a_{i}$, $b_{i},$ and $\beta_{i}$, such that:

(7) $$f_{L}\left(x_{j}\right)=\sum_{i=1}^{K}\beta_{i}G(a_{i},b_{i},x_{j})=t_{j},\;j=1,2,\ldots ,n$$

where $a_{i}$ and $b_{i}$ respectively denote the weight and bias of the *i*th hidden node that is randomly generated, *K* represents the number of hidden nodes, G denotes the activation function, and $\beta_{i}$ indicates the weight vector connecting the *i*th hidden node to the output nodes. Then, we can write Equation (7) compactly as follows:

(8) Hβ=T

where

(9) $$\begin{array}{c} H=\left[\begin{array}{c} h(x_{1})\\ \vdots \\ h(x_{n}) \end{array}\right]=\left[\begin{array}{ccc} G(a_{1},b_{1},x_{1}) & \cdots & G(a_{K},b_{K},x_{1})\\ \vdots & \ddots & \vdots \\ G(a_{1},b_{1},x_{n}) & \cdots & G(a_{K},b_{K},x_{n}) \end{array}\right]\\ \beta =\left[\begin{array}{c} \beta_{1}\\ \vdots \\ \beta_{K} \end{array}\right],\;T=\left[\begin{array}{c} t_{1}\\ \vdots \\ t_{K} \end{array}\right] \end{array}$$

Since H and T are both known, β can be directly calculated as follows:

(10) $$\hat{\beta }=H^{†}T$$

where $H^{†}$ denotes the Moore–Penrose generalized inverse of the hidden layer output matrix H. ELM can be also trained in the viewpoint of optimization, i.e., simultaneously minimizing ${\left\|H\beta -T\right\|}^{2}$ and ${\left\|\beta \right\|}^{2}$. Then, the issue can be described as follows:

(11) $$\begin{array}{l} Minimize:\frac{1}{2}{\left\|\beta \right\|}^{2}+\frac{1}{2}C{\sum_{i=1}^{n}\left\|\xi_{i}\right\|}^{2}\\ Subjectto:h\left(x_{i}\right)\beta =t_{i}-\xi_{i},\;i=1,\;2,\ldots ,n\; \end{array}$$

where $\xi_{i}$ denotes the training error of the training instance $x_{i}$ and *C* represents the penalty factor, which is the tradeoff between training errors and the generalization ability of the learning model. Then, β can be solved as:

(12) $$\beta =\left\{\begin{array}{c} H^{T}{\left(\frac{I}{C}+HH^{T}\right)}^{-1}T,\;\mathrm{when}\;n\leq K\\ {\left(\frac{I}{C}+HH^{T}\right)}^{-1}H^{T}T,\;\mathrm{when}\;n>K \end{array}\right.$$

However, ELM treats the training error of each instance equally, which can cause the learning model to be partial to the majority class when the data distribution is biased. WELM addresses this issue by introducing a weighted matrix W into the optimization formula described in Equation (11), which is rewritten as follows:

(13) $$\begin{array}{c} Minimize:\frac{1}{2}{\left\|\beta \right\|}^{2}+\frac{1}{2}CW{\sum_{i=1}^{n}\left\|\xi_{i}\right\|}^{2}\\ Subject\;to:h\left(x_{i}\right)\beta =t_{i}-\xi_{i},\;\;i=1,\;2,\ldots ,n\; \end{array}$$

where W is a n×n diagonal matrix in which each value on its diagonal represents the corresponding regulation weight of the penalty factor *C*. In [35], W was suggested to be set as:

(14) $$W_{ii}=\left\{\begin{array}{c} \frac{1}{\sum_{j=1}^{n}{IN(t}_{j}=1)},\;if\;t_{i}=1\;\\ \frac{1}{\sum_{j=1}^{n}{IN(t}_{j}=-1)},\;if\;t_{i}=-1 \end{array}\right.$$

where IN is the indicator function, which returns 1 if the corresponding condition holds; otherwise, it returns 0. Then, the solution of β is represented as:

(15) $$\beta =\left\{\begin{array}{c} H^{T}{\left(\frac{I}{C}+WHH^{T}\right)}^{-1}WT,\;\mathrm{when}\;n\leq K\\ {\left(\frac{I}{C}+HWH^{T}\right)}^{-1}H^{T}WT,\;\mathrm{when}\;n>K \end{array}\right.$$

By means of this weighting strategy, the training errors of the minority instances are exerted larger penalties than those of the majority class, further providing an impartial training result.

### 2.4. Description of the OMAL Algorithm

Next, the procedure of the OMAL algorithm (Algorithm 1) is described as follows:
**Algorithm 1:** OMAL**Input:** a null labeled set $\Phi_{L}$, an unlabeled multi-label data block stream $u_{1},u_{2},\ldots ,\;u_{t},\ldots$, the significance level of query strategy regulation weight λ, the querying rate θ, the activation function *G*, the number of hidden nodes *K*, and the penalty factor *C*.**Output:** the current classification model *S*.**Procedure:**Label $u_{1}$ by the oracle when we have received it;Put the labeled $u_{1}$ into $\Phi_{L}$;Remove $u_{1}$;On $\Phi_{L}$, for each label *i* = 1, 2,…,|*L*|, calculate $r_{i}$ by Equation (6);Acquire the label ranking sequence *R* by arranging all $r_{i}$ in descending order;For each label in *R*, train the corresponding binary classifiers ${CL}_{1},\;{CL}_{2},\;\ldots ,\;{CL}_{\left|L\right|}$ by calling WELM;Acquire the initial CC classification model *S* = [${CL}_{1},\;{CL}_{2},\;\ldots ,\;{CL}_{\left|L\right|}$];When a new unlabeled data chunk $u_{t}$ is received:  Transform the output of each instance in $u_{t}$ on *S* to be posterior probabilities by Equation (1);  Calculate the average entropy of each instance in $u_{t}$ by Equation (2);  Calculate the diversity level of each instance in $u_{t}$ by Equations (3) and (4);  Calculate the significance level of each instance in $u_{t}$ by Equation (5);  Rank the significance level of all instances in $u_{t}$ in descending order and further select the first θ instances from them for querying;   Label these selected instances by the oracle;  Add the new labeled instances to $\Phi_{L}$;   Remove $u_{t}$;   On $\Phi_{L}$, for each label *i* = 1, 2,…,|*L*|, calculate $r_{i}$ by Equation (6);  Acquire the label ranking sequence *R* by arranging all $r_{i}$ in descending order;   For each label in *R*, train the corresponding binary classifiers ${CL}_{1},\;{CL}_{2},\;\ldots ,\;{CL}_{\left|L\right|}$ by calling WELM;  Update the CC classification model *S* by the newly trained |*L*| binary classifiers, i.e., *S* = [${CL}_{1},\;{CL}_{2},\;\ldots ,\;{CL}_{\left|L\right|}$];  Output the current classifier *S*;  Return to step 8.

Specifically, the querying rate θ∈[0%,100%] is a user-specified parameter that is used to decide how many instances should be selected from an unlabeled block for querying. An undersize θ tends to miss some significant instances, which may help to improve classification performance, while an oversize θ tends to introduce some useless information while increasing the labeling burden and training time. In next section, the impact of θ will be presented by describing the experimental results in detail.

In addition, it can be observed that OMAL fails to consider concept drift [46], which may emerge in multi-label data streams. In such a scenario, it is suggested that LD3 [39] can be first used to detect whether concept drift has happened, and if it has, its strength should be further measured. Then, the labeled instances with time stamps should be refined based on the estimated concept drift strength. This means that some older labeled instances should be removed from the labeled set $\Phi_{L}$ to avoid their participation in training new classification models. Such manipulation can be seen as an alternative of the forgetting mechanism.

### 2.5. Time Complexity of the OMAL Algorithm

Finally, we try to analyze the time complexity of the proposed OMAL algorithm running on a round. First, both the transformation of posterior probabilities and the calculation of average entropy consume *O*(*k*) time. The time complexity of calculating the average diversity is *O*(*nk*). Then, calculating the significance levels of all unlabeled instances in the current block $u_{t}$ consumes *O*(*k*) time. Next, generating the co-occurrence matrix costs *O*(*n|L|*) time, calculating the global rankings consumes *O*(*|L|*^2^) time, and ranking the label sequence takes *O*(*|L|*log*|L|*) time. As we know, the time complexity of training a WELM classifier is *O*(*n*^3^), and thus, training |*L*| WELMs in CC costs *O*(*n*^3^*|L|*) in total. In general, *k* << *n* and *|L|* << *n*^3^, and thus, the time complexity of OMAL is *O*(*n*^3^*|L|*). In other words, training binary classifiers dominates the time consumption of OMAL.

Therefore, there exist two potential risks that hinder the application of OMAL in some scenarios that have high demands for running time. The first one is that with a continuous increase in labeled instances, the time burden of updating classifiers will increase in exponential fashion. While the second one is that if facing a dataset with tremendous labels, the time consumption may become unacceptable. In such scenarios, we suggest using incremental multi-label learning models, which do not require revisiting old labeled instances, and, at the same time, can focus on label correlations to some extent and dynamically adapt to the variance in class imbalance, e.g., LW-ELM [47], to replace the combination of CC and WELM. Although such a replacement may have some negative impacts on the classification performance of learning models, but it can still be regarded as an effective alternative to maintain the tradeoff between model performance and running time.

## 3. Results

### 3.1. Datasets Used in This Study

In this study, all experiments were conducted on ten multi-label datasets acquired from the Multi-Label Classification Dataset Repository, which is available at http://www.uco.es/kdis/mllresources/ (accessed on 17 December 2024). These datasets contain 194~10,810 instances, 16~1449 features, 5~45 labels, and 1.074~5.073 label cardinality (LC), which indicates the average number of labels on each instance, and cover various fields, including image, medicine, music, chemistry, biology, and text. Specifically, these datasets were randomly transformed to be data streams with different block sizes, and for each dataset, 30% of the instances were reserved as testing instances. A detailed description about these datasets is presented in Table 1.

### 3.2. Experimental Settings

All experiments were conducted in the environment of Python 3.8 on AMD Ryzen 9 7945HX with Radeon Graphics 2.50 GHz and 32 GB RAM. We compared the proposed OMAL algorithm with a baseline query algorithm, i.e., Random, and three previous algorithms, including EMAL [18], LCI [19], and CVIRS [20]. Specifically, Random extracts unlabeled instances randomly for submission to the oracle for labeling. To guarantee the impartiality of the comparative experiments, all algorithms used CC [29,30] as the basic multi-label classifier, where WELM [35] was adopted as the basic binary classifier. In addition, the grid search strategy was used to determine the best combination of parameters *K* and *C* by an internal five-fold cross validation, in which K∈{100,200,….,2000} and $C\in \{10^{-3},10^{-2},\ldots ,10^{5}\}$, respectively.

As for the performance evaluation metrics, we used the two most popular metrics for evaluating the quality of a multi-label learning algorithm considering imbalanced data distributions, which are the Macro-F1 and Micro-F1 metrics [48]. Both Macro-F1 and Micro-F1 evaluate the harmonic means of precision and recall, and they can be defined as follows:

(16) $$\mathrm{Macro}-F1=\frac{2\times P^{\mathrm{macro}\;}\times R^{\mathrm{macro}\;}}{P^{\mathrm{macro}\;}+R^{\mathrm{macro}\;}}\;$$

(17) $$\mathrm{Micro}-F1=\frac{2\times P^{\mathrm{micro}\;}\times R^{\mathrm{micro}\;}}{P^{\mathrm{micro}\;}+R^{\mathrm{micro}\;}}$$

where $P^{\mathrm{macro}\;}$ and $R^{\mathrm{macro}\;}$ represent, respectively, macroscopic precision and recall, and they are defined as follows:

(18) $$P^{\mathrm{macro}\;}=\frac{1}{\left|L\right|}\sum_{i=1}^{\left|L\right|}\;\frac{TP_{i}}{TP_{i}+FP_{i}}$$

(19) $$R^{\mathrm{macro}\;}=\frac{1}{\left|L\right|}\sum_{i=1}^{\left|L\right|}\;\frac{TP_{i}}{TP_{i}+FN_{i}}$$

where $TP_{i}$, $FP_{i}$, and $FN_{i}$ denote the number of true positive, false positive, and false negative instances on the *i*th label predicted by the classification model, respectively. $P^{\mathrm{micro}\;}$and $R^{\mathrm{micro}\;}$ respectively denote the microscopic precision and recall, which can be calculated as follows:

(20) $$P^{\mathrm{micro}\;}=\frac{\sum_{i=1}^{\left|L\right|}\;TP_{i}}{\sum_{i=1}^{\left|L\right|}\;\left(TP_{i}+FP_{i}\right)}$$

(21) $$R^{\mathrm{micro}\;}=\frac{\sum_{i=1}^{\left|L\right|}\;TP_{i}}{\sum_{i=1}^{\left|L\right|}\;\left(TP_{i}+FN_{i}\right)}$$

Furthermore, considering the randomness of data streams, each experiment was randomly conducted twenty times, and then, the average performance was presented in the corresponding learning curve. The area under learning curve (ALC) was finally calculated to compare the quality of the various active learning algorithms.

### 3.3. Experimental Results

**Figure 3:**
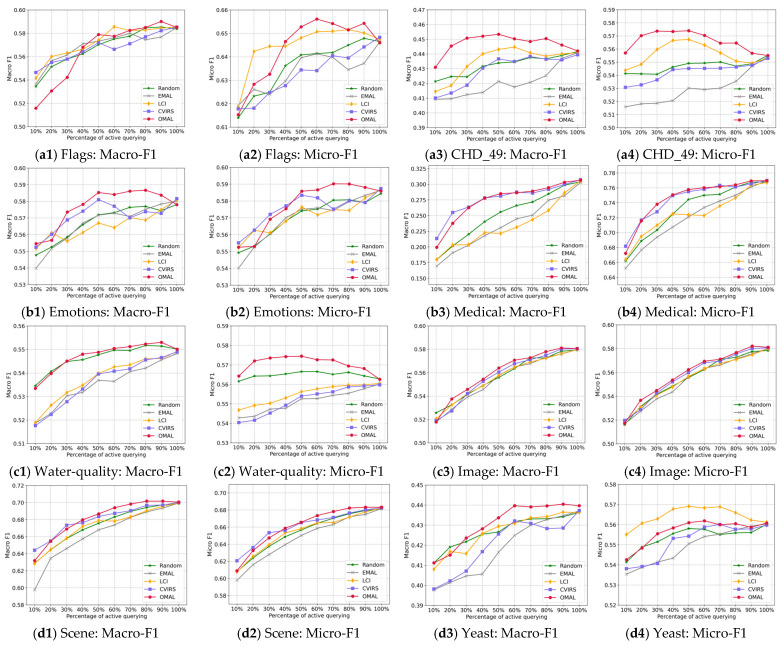

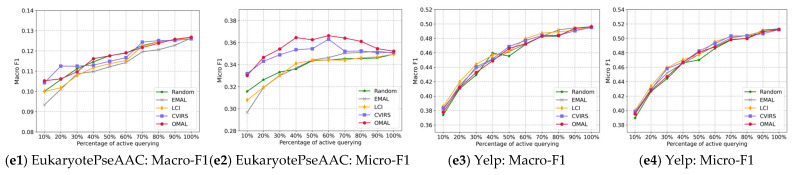
Best performance variance of the various algorithms with the variance in the parameter θ on the various datasets.

First, we varied the querying rate θ from 10% to 100% with an increment of 10% to observe the impact of this parameter on the learning effect. Figure 3 presents the trends of the various active learning algorithms with the variance of θ on each dataset.

The results in Figure 3 show the following:

(1) When increasing the value of θ, i.e., actively querying more unlabeled instances, the various active learning algorithms tend to yield better classification performance;
(2) When θ is designated as a small value, increasing it can provide a more significant performance improvement;
(3) After designating a medium value for θ, continually increasing the value of θ can not significantly improve classification performance, and even on some datasets, the performance presents a declining trend.

It is not difficult to understand the first phenomenon, where, although active learning can provide the most informative instances, if they are extremely scare, it still tends to yield underfitting results for classification models. The two other phenomena tell us that when a sufficient number of informative instances have been learned, it is not necessary to continually learn the remaining instances; otherwise, more noise will be added, and more labeling burdens will be required. According to the feedback from the results in Figure 3, we empirically set θ=60%.

**Figure 4:**
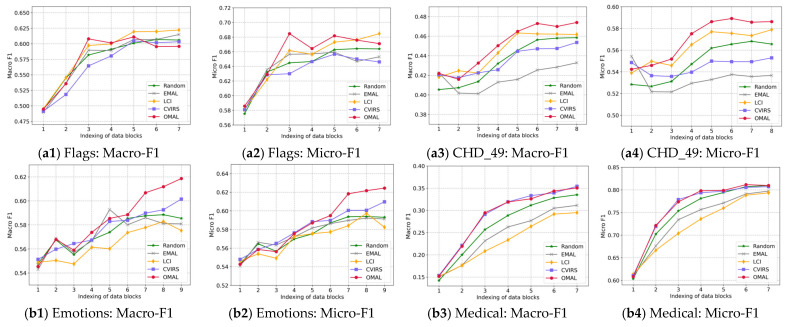

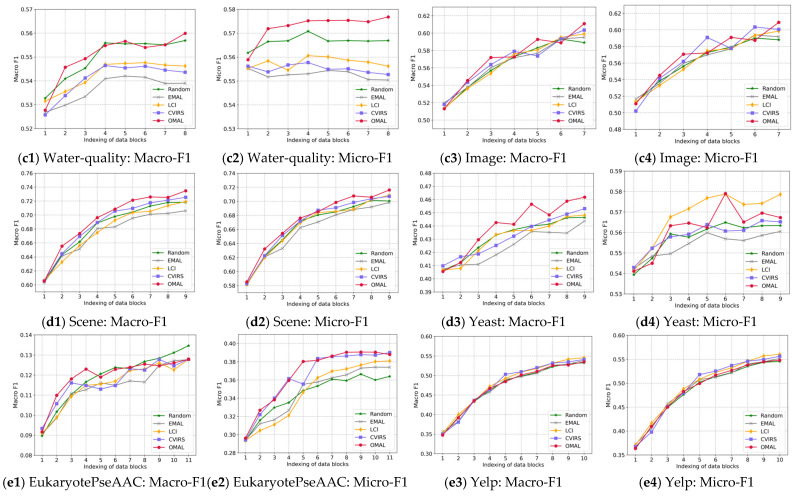
Learning curve of each active learning algorithm on each dataset.

Next, we present the learning curves of several comparable algorithms on each data stream in Figure 4. Furthermore, the ALC values and average rankings of various algorithms based on the Macro-F1 and Micro-F1 metrics are shown in Table 2 and Table 3, respectively.

From the results in Figure 4 and Table 2 and Table 3, we can draw several conclusions, as follows:

(1) In comparison to Random, several of the active learning algorithms except EMAL yielded higher ALC values in terms of both the Macro-F1 and Micro-F1 metrics, indicating that these algorithms can select more significant unlabeled instances to query and further help to improve the quality of a multi-label learning model to a large extent. As the worst querying strategy, EMAL only focuses those instances that are closest to the classification boundary, but it neglects the diversity of the data distribution, which tends to make the classification boundary converge to a local optimum one. This explains why EMAL performs worse than Random.
(2) Although LCI acquired a higher average ranking than Random on both metrics, its superiority is not significant enough. In essence, LCI can be seen as an unconventional query strategy with partial exploration ability, as it always queries those unlabeled instances with a significant difference in terms of label cardinality with labeled instances. Therefore, we cannot prevent it from converging to a local optimum boundary rapidly, but its convergence speed is obviously slower than EMAL.
(3) Both CVIRS and the proposed OMAL significantly outperform several of the other algorithms. It is difficult to understand this result since both these algorithms simultaneously focus on the significance of unlabeled instances in terms of both *uncertainty* and *diversity*. Therefore, we can say that CVIRS and OMAL both own exploitation and exploration abilities. More informative instances can be selected for querying by them.
(4) In comparison to CVIRS, OMAL adopts a direct way of exploring *diversity* in the feature space, which helps to rapidly adapt the potential variance in the feature space in the data stream. In contrast, the exploration of *diversity* by observing the variance in the label vectors adopted by CVIRS may be not robust enough. In addition, the time consumption of CVIRS is always higher than that of OMAL, owing to the sophisticated ranking aggregation strategy adopted by CVIRS.
(5) OMAL yielded the best ALC value based on the Macro-F1 metric on seven datasets and the best ALC value based on the Micro-F1 metric on eight datasets. In addition, OMAL acquired the lowest average ranking of 1.4 on both metrics. These results show its effectiveness and superiority in dealing with multi-label data stream active learning scenarios. We believe that on a static unlabeled set, where all instances are initially available, the continuously strong space exploration ability of OMAL may be not necessary. While on a dynamic data stream, it will contribute more to the performance improvement of the classification model.

### 3.4. Significance Analysis

**Figure 5:**
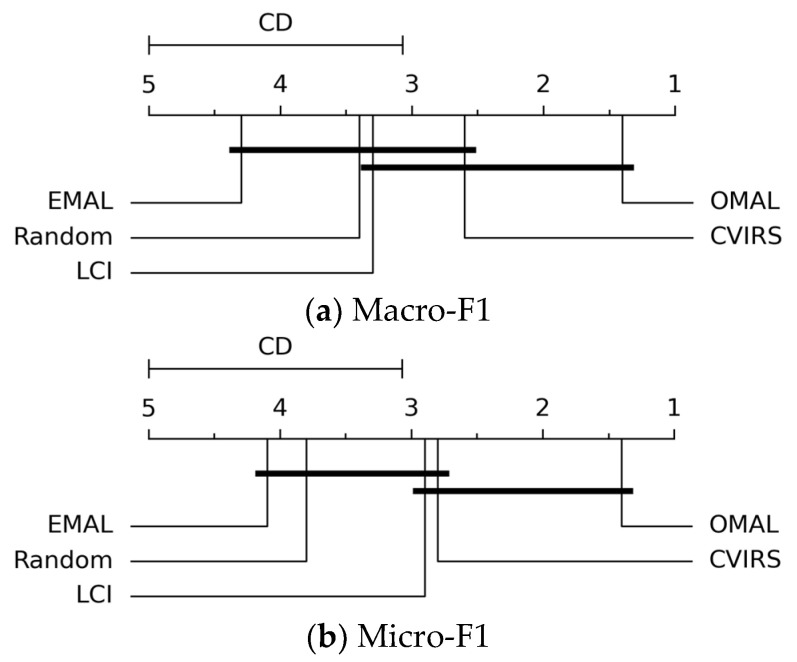
CD diagrams at the standard level of significance α=0.05 in terms of both the Macro-F1 and Micro-F1 metrics.

Next, the Nemenyi test, a post hoc test for the Friedman test [49,50], was adopted to observe whether there exists a significant difference between the proposed OMAL algorithm and any one of the compared algorithms. Specifically, if the average ranking of OMAL and that of a compared algorithm differ by at least one critical difference (CD) unit, then we considered that their performances are significantly different. Specifically, the CD was calculated as follows:

(22) $$CD=q_{\alpha }\sqrt{\frac{J(J+1)}{6D}}$$

where $q_{\alpha }$ denotes the significance level, *J* represents the number of compared algorithms, and *D* indicates the number of datasets. In our experiments, *J* = 5 and *D* = 10, and thus, with the significance level α=0.05, we calculate CD=1.9288 by Equation (22). The CD diagrams are presented in Figure 5.

In Figure 5, we observe that on both metrics, our proposed OMAL algorithm significantly outperforms both the EMAL and Random algorithms. While in comparison with the LCI and CVIRS algorithms, although the superiority of OMAL is not significant, it still acquires an obviously lower average ranking than them.

### 3.5. Ablation Experiments

Next, we conducted three groups of ablation experiments to estimate whether the adoption of some components or strategies are necessary.

In the first group of ablation experiments, we focused on the query strategy. Specifically, we compared the performance of only adopting the *uncertainty* query criterion, only adopting the *diversity* query criterion, and integrating both. The results in Table 4 show that integrating both *uncertainty* and *diversity* is necessary as it yielded the best results on seven and eight datasets in terms of the Macro-F1 and Micro-F1 metrics, respectively. This means that the local search ability and the global exploration ability are both very important for active learning. In addition, we note that using only *uncertainty* as the querying strategy seems to easily yield a better performance than using only *diversity*. However, this does not mean that actively querying *diversity* instances is useless.

In the second group of ablation experiments, we compared ELM and WELM further to judge whether the class imbalance issue should be considered in multi-label active learning. The results in Table 5 illustrate that WELM yielded the best results on nine and eight datasets, which is significantly superior to ELM, indicating it is necessary to use a classifier that is specifically designed for dealing with the class imbalance issue.

The third group of ablation experiments refers to the label sequence in CC. We compared the performance of CC based on a random label sequence and ranking labels by the significance estimation of LD3. The results in Table 6 indicate that it is necessary to rank the order of labels according to their correlation significance because, on most datasets, adopting the random label sequence yielded a poorer performance than that based on LD3. This means that putting high correlation labels in front of the label sequence of CC can provide more information for improving the classification performance of sequent binary classifiers.

### 3.6. Results of Running Time

Finally, we also compared the average running time of several active learning algorithms throughout all rounds. The results in Table 7 indicate that OMAL is a time-saving algorithm as it always costs an approximately equal running time as the Random, EMAL, and LCI algorithms, but it costs significantly less running time than CVIRS. Considering in our experimental framework, training classifiers dominate the time complexity of several of the algorithms except CVIRS, and thus, the differences in the running time among several active learning algorithms can be ignored. For OMAL, the difference in the number of training instances *n* and the number of labels |*L*| on different datasets result in the difference in the running time.

## 4. Conclusions

This study proposed an active learning algorithm called OMAL, aiming to improve the performance of multi-label learning models in a data stream scenario. Specifically, OMAL adopts a novel integrated query strategy, where the average entropy of prediction probabilities is used to estimate the uncertainty level of unlabeled instances, and the average cosine distance in feature space is used to estimate its diversity level. To focus the label correlations, CC is used as the basic multi-label classifier. Also, to adapt the naturally imbalanced data distribution existing in multi-label data, WELM is used as the basic binary classifier. The experimental results on ten transformed multi-label data streams illustrated that OMAL outperforms random querying and several traditional static active querying algorithms, indicating its effectiveness and superiority. In addition, our ablation experiments verified the significance of each component embedded in OMAL.

Specifically, the contributions of this study can be summarized as follows:

1. It proposed a novel multi-label active query strategy that simultaneously satisfies the requirements of exploration and exploitation in online environments;
2. It designed an effective multi-label online solution to simultaneously leverage label correlation information and adapt the class imbalance distribution;
3. It introduced LD3 label correlation information into a CC model to avoid error accumulation;
4. It presented a subtle multi-label online active learning algorithm that can produce excellent performance and, meanwhile, is relatively time-saving.

In future work, we expect to develop more efficient active learning algorithms for adapting high-speed multi-label data streams. In addition, methods to improve OMAL for tracking and adapting drifting multi-label data streams will also be investigated.

## Figures and Tables

**Table 1:** Datasets used in this study.

Dataset	Domain	#Instances	#Features	#Labels	LC	Block Size
Flags	Image	194	19	7	3.392	20
CHD_49	Medicine	555	49	6	2.580	50
Emotions	Music	593	72	6	1.868	50
Medical	Text	978	1449	45	1.245	100
Water quality	Chemistry	1060	16	14	5.073	100
Image	Image	2000	294	5	1.236	200
Scene	Image	2407	294	6	1.074	200
Yeast	Biology	2417	103	14	4.237	200
EukaryotePseAAC	Biology	7766	440	22	1.146	500
Yelp	Text	10,810	671	5	1.638	800

#Instances, #Features, and #Labels denote the number of instances, features, and labels included in the corresponding dataset.

**Table 2:** ALC values and average ranking of several comparable algorithms based on Macro-F1 metric.

Dataset	Random	EMAL	LCI	CVIRS	OMAL
Flags	0.5751 ± 0.0159	0.5762 ± 0.0064	**0.5856 ± 0.0061**	0.5663 ± 0.0089	0.5774 ± 0.0120
CHD_49	0.4346 ± 0.0067	0.4176 ± 0.0092	0.4447 ± 0.0065	0.4349 ± 0.0066	**0.4503 ± 0.0040**
Emotions	0.5732 ± 0.0100	0.5729 ± 0.0061	0.5642 ± 0.0070	0.5771 ± 0.0106	**0.5841 ± 0.0067**
Medical	0.2661 ± 0.0076	0.2449 ± 0.0054	0.2315 ± 0.0078	**0.2875 ± 0.0030**	0.2865 ± 0.0031
Water-quality	0.5497 ± 0.0023	0.5365 ± 0.0015	0.5426 ± 0.0026	0.5408 ± 0.0024	**0.5504 ± 0.0025**
Image	0.5636 ± 0.0059	0.5649 ± 0.0070	0.5648 ± 0.0042	0.5678 ± 0.0051	**0.5708 ± 0.0032**
Scene	0.6832 ± 0.0037	0.6738 ± 0.0033	0.6782 ± 0.0058	0.6876 ± 0.0031	**0.6940 ± 0.0034**
Yeast	0.4318 ± 0.0051	0.4247 ± 0.0033	0.4309 ± 0.0043	0.4321 ± 0.0024	**0.4397 ± 0.0027**
EukaryotePseAAC	0.1189 ± 0.0025	0.1143 ± 0.0008	0.1153 ± 0.0005	0.1167 ± 0.0018	**0.1193 ± 0.0013**
Yelp	0.4716 ± 0.0025	0.4722 ± 0.0041	**0.4796 ± 0.0021**	0.4771 ± 0.0021	0.4726 ± 0.0013
Average ranking	3.4	4.3	3.3	2.6	1.4

All results are presented in the form of mean ± standard deviation, and the best one for each dataset is highlighted in bold.

**Table 3:** ALC values and average rankings of several comparable algorithms based on the Micro-F1 metric.

Dataset	Random	EMAL	LCI	CVIRS	OMAL
Flags	0.6414 ± 0.0103	0.6413 ± 0.0058	0.6507 ± 0.0054	0.6341 ± 0.0071	**0.6561 ± 0.0041**
CHD_49	0.5493 ± 0.0076	0.5337 ± 0.0110	0.5630 ± 0.0069	0.5453 ± 0.0078	**0.5704 ± 0.0041**
Emotions	0.5752 ± 0.0104	0.5759 ± 0.0057	0.5718 ± 0.0072	0.5819 ± 0.0105	**0.5866 ± 0.0061**
Medical	0.7501 ± 0.0061	0.7336 ± 0.0034	0.7230 ± 0.0077	0.7587 ± 0.0041	**0.7602 ± 0.0022**
Water quality	0.5666 ± 0.0021	0.5527 ± 0.0012	0.5577 ± 0.0021	0.5551 ± 0.0019	**0.5727 ± 0.0022**
Image	0.5625 ± 0.0058	0.5635 ± 0.0065	0.5633 ± 0.0046	0.5683 ± 0.0046	**0.5695 ± 0.0033**
Scene	0.6644 ± 0.0038	0.6586 ± 0.0032	0.6649 ± 0.0060	0.6684 ± 0.0033	**0.6735 ± 0.0036**
Yeast	0.5577 ± 0.0053	0.5541 ± 0.0023	**0.5683 ± 0.0022**	0.5588 ± 0.0026	0.5619 ± 0.0031
EukaryotePseAAC	0.3442 ± 0.0087	0.3464 ± 0.0014	0.3471 ± 0.0042	0.3631 ± 0.0034	**0.3663 ± 0.0029**
Yelp	0.4861 ± 0.0026	0.4896 ± 0.0036	**0.4955 ± 0.0021**	0.4929 ± 0.0013	0.4881 ± 0.0010
Average ranking	3.8	4.1	2.9	2.8	1.4

All results are presented in the form of mean ± standard deviation, and the best one for each dataset is highlighted in bold.

**Table 4:** Ablation experiment on the querying strategy, in which the best results have been highlighted in bold.

Dataset	Macro-F1			Micro-F1		
	Uncertainty	Diversity	Both	Uncertainty	Diversity	Both
Flags	0.5519 ± 0.0113	0.5762 ± 0.0047	**0.5774 ± 0.0012**	0.6397 ± 0.0060	0.6445 ± 0.0023	**0.6561 ± 0.0041**
CHD_49	0.4494 ± 0.0044	0.4448 ± 0.0040	**0.4503 ± 0.0040**	0.5678 ± 0.0061	0.5601 ± 0.0039	**0.5704 ± 0.0041**
Emotions	**0.5888 ± 0.0073**	0.5808 ± 0.0056	0.5841 ± 0.0067	**0.5927 ± 0.0074**	0.5834 ± 0.0053	0.5866 ± 0.0061
Medical	0.2852 ± 0.0045	0.2777 ± 0.0024	**0.2865 ± 0.0031**	0.7591 ± 0.0043	0.7511 ± 0.0029	**0.7602 ± 0.0022**
Water quality	**0.5557 ± 0.0016**	0.5399 ± 0.0015	0.5504 ± 0.0025	**0.5772 ± 0.0011**	0.5581 ± 0.001	0.5727 ± 0.0022
Image	0.5642 ± 0.0046	0.5656 ± 0.0045	**0.5708 ± 0.0032**	0.5629 ± 0.0041	0.5643 ± 0.0046	**0.5695 ± 0.0033**
Scene	0.6922 ± 0.0031	0.6786 ± 0.0027	**0.6940 ± 0.0034**	0.6731 ± 0.0029	0.6582 ± 0.003	**0.6735 ± 0.0036**
Yeast	0.4351 ± 0.0029	0.4350 ± 0.0013	**0.4397 ± 0.0027**	0.5585 ± 0.0021	0.5497 ± 0.0009	**0.5619 ± 0.0031**
EukaryotePseAAC	**0.1276 ± 0.0014**	0.1124 ± 0.0008	0.1193 ± 0.0013	0.3461 ± 0.0021	0.3642 ± 0.0012	**0.3663 ± 0.0029**
Yelp	0.4678 ± 0.0013	0.4718 ± 0.0014	**0.4726 ± 0.0013**	0.4838 ± 0.0013	0.4866 ± 0.0020	**0.4881 ± 0.0010**

**Table 5:** Ablation experiment on considering the class imbalance issue, in which the best results have been highlighted in bold.

Dataset	Macro-F1		Micro-F1	
	ELM	WELM	ELM	WELM
Flags	0.5766 ± 0.0159	**0.5774 ± 0.0012**	**0.6682 ± 0.0118**	0.6561 ± 0.0041
CHD_49	0.4454 ± 0.0058	**0.4503 ± 0.0040**	**0.5855 ± 0.0074**	0.5704 ± 0.0041
Emotions	0.5742 ± 0.0079	**0.5841 ± 0.0067**	0.5765 ± 0.0081	**0.5866 ± 0.0061**
Medical	0.2805 ± 0.0052	**0.2865 ± 0.0031**	0.7194 ± 0.0055	**0.7602 ± 0.0022**
Water quality	0.4822 ± 0.0097	**0.5504 ± 0.0025**	0.5415 ± 0.0057	**0.5727 ± 0.0022**
Image	0.4461 ± 0.0060	**0.5708 ± 0.0032**	0.4498 ± 0.0059	**0.5695 ± 0.0033**
Scene	0.5965 ± 0.0036	**0.6940 ± 0.0034**	0.5769 ± 0.0041	**0.6735 ± 0.0036**
Yeast	0.4023 ± 0.0031	**0.4397 ± 0.0027**	0.5601 ± 0.0040	**0.5619 ± 0.0031**
EukaryotePseAAC	0.0877 ± 0.0031	**0.1193 ± 0.0013**	0.2735 ± 0.0016	**0.3663 ± 0.0029**
Yelp	**0.4771 ± 0.0037**	0.4726 ± 0.0013	0.4871 ± 0.0034	**0.4881 ± 0.0010**

**Table 6:** Ablation experiment on the label ranking strategy in CC, in which the best results have been highlighted in bold..

Dataset	Macro-F1		Micro-F1	
	Random	LD3	Random	LD3
Flags	0.5764 ± 0.0077	**0.5774 ± 0.0012**	0.6553 ± 0.0071	**0.6561 ± 0.0041**
CHD_49	**0.4526 ± 0.0046**	0.4503 ± 0.0040	0.5692 ± 0.0085	**0.5704 ± 0.0041**
Emotions	0.5791 ± 0.0093	**0.5841 ± 0.0067**	0.5802 ± 0.0121	**0.5866 ± 0.0061**
Medical	0.2809 ± 0.0027	**0.2865 ± 0.0031**	0.7547 ± 0.0041	**0.7602 ± 0.0022**
Water quality	0.5487 ± 0.0077	**0.5504 ± 0.0025**	**0.5782 ± 0.0054**	0.5727 ± 0.0022
Image	0.5671 ± 0.0060	**0.5708 ± 0.0032**	0.5662 ± 0.0053	**0.5695 ± 0.0033**
Scene	0.6916 ± 0.0033	**0.6940 ± 0.0034**	**0.6748 ± 0.0039**	0.6735 ± 0.0036
Yeast	0.4386 ± 0.0060	**0.4397 ± 0.0027**	0.5562 ± 0.0179	**0.5619 ± 0.0031**
EukaryotePseAAC	0.1132 ± 0.0051	**0.1193 ± 0.0013**	0.2855 ± 0.0319	**0.3663 ± 0.0029**
Yelp	0.4712 ± 0.0029	**0.4726 ± 0.0013**	0.4841 ± 0.0042	**0.4881 ± 0.0010**

**Table 7:** Average running time of each active learning algorithm throughout all rounds (seconds).

Dataset	Random	EMAL	LCI	CVIRS	OMAL
Flags	0.0409	0.0464	0.0483	0.3152	0.0443
CHD_49	0.1646	0.1758	0.1665	2.2945	0.1687
Emotions	0.4059	0.4260	0.4271	2.7905	0.4117
Medical	8.0610	8.3567	8.1836	14.7187	8.4605
Water quality	0.6095	0.6342	0.7039	8.2412	0.6541
Image	1.0262	1.0604	1.1134	26.6437	1.1383
Scene	3.2678	3.5351	3.5442	40.3567	3.5444
Yeast	8.4641	8.8564	8.7347	46.9647	9.1096
EukaryotePseAAC	109.7874	110.9367	111.7936	483.9314	109.8256
Yelp	34.0957	34.7724	34.1290	780.7408	35.3902

## Data Availability

All multi-label datasets used in the experiments are from a multi-label classification dataset repository that is available at http://www.uco.es/kdis/mllresources/ (accessed on 17 December 2024).

## Abbreviations

The following abbreviations are used in this manuscript:

ALC	area under learning curve
CC	classifier chain
CD	critical difference
CVIRS	category vector inconsistency and ranking of scores
ELM	extreme learning machine
EMAL	example-based active learning
KNN	K-nearest neighbors
LC	label cardinality
LCI	label cardinality inconsistency
LD3	label dependency drift detector
OMAL	online multi-label active learning
WELM	weighted extreme learning machine
